# Patterns of adult and youth inpatient admissions before and after the COVID-19 pandemic in a psychiatric ward: an observational study

**DOI:** 10.1186/s12913-022-08374-8

**Published:** 2022-08-17

**Authors:** Carolina Alves Rodrigues, Nuno Rodrigues, Miguel Nascimento, Joana Oliveira-Silva

**Affiliations:** 1 Centro Hospitalar Psiquiátrico de Lisboa, Avenida do Brasil 53, 1749-002 Lisbon, Portugal; 2 Hospitais da Universidade de Coimbra, Praceta Professor Mota Pinto, 3004-561 Coimbra, Portugal

**Keywords:** COVID-19, Psychiatric Department, Delivery of Health Care, Mental disorders, Patient Readmissions

## Abstract

**Background:**

The current pandemic situation led to deep changes not only in social relationships, but also on clinical care and access to healthcare facilities. The authors aimed to understand whether this context affected the main characteristics of psychiatric hospitalizations, measured by admissions in a portuguese acute psychiatric ward.

**Methods:**

Retrospective data collection of all patients admitted in Centro Hospitalar Psiquiátrico de Lisboa, in two different time periods: pre-COVID-19 (march 11th, 2019 to march 10th, 2020, *n* = 1845) and COVID-19 (march 11th, 2020 to march 10th, 2021, *n* = 1278); comparing the number of total admissions, compulsory ones, age, sex, median days of admission, median days to readmission and diagnosis at discharge. Distribution of disorders in both groups, as well as in compulsory admissions were also evaluated. The same comparisons were evaluated in the 15–25-year-old patient group.

**Results:**

Statistical significance was found regarding total number of admissions (reduction of around 30.7%), as well as compulsory ones (reduction of 14%, although the relative frequency had increased), days of admission and distribution between admissions (with lower reductions regarding dementias, schizophrenia and affective disorders, while substance use disorders and intellectual disabilities presented reductions of over 50%), with no differences between gender, median age, previous admissions or readmissions. Distribution between compulsory admissions did not present differences before and during COVID periods.

For patients between 15 and 25 years of age, statistical significance was found regarding total number of compulsory ones (94 versus 44, *p*-value = 0.01), and in all groups of diagnoses (all with *p*-value = 0.001).

**Conclusions:**

While there was a general reduction in the overall number of patients admitted, in the most severe conditions (compulsory admissions and schizophrenia diagnosis) did not present such a reduction. Difficulties in social, clinical and family networks can explain the reduction of the time to readmission. Future research could show whether there is a rebound increase number of admissions in the other diagnoses.

**Trial Registration:**

The current study was approved by the hospital’s scientific and ethics committees (CCP number 0060/2021 and CES 09/2021).

## Background

The spread of SARS-CoV-2, the virus that ended up causing the 2019 coronavirus disease (COVID-19) and pandemic implicated a drastic shift in our patterns of living. The COVID-19 outbreak was declared an international public health emergency on January 30, 2020 by the World Health Organization (WHO) [1]. Authorities in various countries soon enforced strict measurements of social distancing, such as implementing online labour, the closing of schools and cancelation of major public events and limiting contact outside the family core. Although these had the purpose of ensuring safety and limiting the spread of the virus, they imposed disruption in people’s lives. The unpredictability and rapidness of the spreading of the disease and its impact in daily life has been causing universal awareness, along with anxiety and distress, all of which are considered natural responses to the randomly changing circumstances [2].

According to a nationwide study on mental health [3], more than 20% of the Portuguese population has been diagnosed with mental illness up to 2013. The most prevalent disorders were anxiety (16,5%) and depressive (7,9%) disorders. The estimated lifetime prevalence of mental disorders in the country is 42,7%, which is much higher than the rest of the world (29.2%) [4]. Considering the impact of the COVID-19 pandemic in the daily life of Portuguese people, this could mean an increase in the incidence of psychiatric illnesses after the emergence of the SARS-CoV-2, as well as a change in the patterns of previously diagnosed patients admitted to psychiatric wards during the pandemic. The purpose of this study is to ascertain whether the main characteristics of the patients admitted to a psychiatric ward were affected during the pandemic period, and to ultimately reflect on the fragilities of a health care system which was put under extreme strain. This contemplation becomes even more relevant through the severe mental illness (SMI) optics, which usually reflects the functional impairments caused by mental disease, and is commonly associated with certain types of diagnosis, such as psychotic disorders or schizophrenia, but also to number of hospital admissions and even days of hospitalization in previous years [5].

### The psychosocial impact of COVID-19

COVID-19 required the implementation of quarantine measures as a fundamental disease control measure. In 2003, during the severe acute respiratory syndrome coronavirus 1 (SARS) pandemic, which was also successfully contained through the similar measures, one study showed that a substantial proportion of quarantined persons felt distressed. This was displayed by a high proportion of post-traumatic stress disorder (PTSD) and depressive symptoms manifested in this population. In the same study, the presence of these symptoms was highly correlated with the duration of the quarantine [2]. In a sample of 838 hospital staff members, there were reports of higher likeliness of exhaustion, detachment from others, anxiety when dealing with febrile patients, irritability, insomnia, poor concentration and indecisiveness, deteriorating work performance and reluctance to work or consideration of resignation immediately after a quarantine period [6]. Besides quarantine measures, COVID-19 infection itself has been studied as a potential risk factor for the development of psychiatric symptoms. In a systematic review published in 2021, elevated rates of depression, anxiety, fatigue and sleep difficulties were reported in COVID-19 survivors [7]. In a primary cohort of 236,379 patients diagnosed with COVID-19, a hazard ratio of risk for psychiatric disorder diagnosis higher than 1 was detected immediately after the infection and, although declining, it remained significantly high 6 months after COVID-19 diagnosis [8]. This data should help the conclusion that COVID-19 infection and possibly quarantine measures may pose as a personalized trauma and a risk factor for those who go through the process, with possible short and long-time effects on mental health and psychiatric admissions.

### Impact on people with pre-existing psychiatric illness and SMI

Evidence shows that people with psychiatric illness may be more prone to COVID-19 infection [9]. A number of factors may be involved in the increased susceptibility for infection, such as suboptimal behaviours and life style choices (smoking), socioeconomic status, cognitive deficits, poor awareness level, impaired risk perception and reduced concern about hygiene [9–12]. Patients with mental health conditions may be substantially influenced by the emotional responses brought on by the COVID-19 pandemic, which may result in worsening of already existing symptoms or full relapses with need for further care. For example, individuals with known obsessive compulsive disorders (OCD) may practice frequent self-monitoring of body temperature to check for fever or increase behaviours such as frequent hand-washing [1]. People with high health anxiety, who tend to perceive bodily sensations or changes (e.g., fever, coughing, aching muscles) as symptoms of being ill are also more likely to present higher levels of anxiety, either avoiding hospitals and doctors’ offices altogether, by perceiving them as possible sources of contagion, or engage in multiple visits to doctors and emergency rooms in pursuit of reassurance of their beliefs [13]. When it comes to SMI, which may be characterized by an impairment in insight and decision-making capacity, it could complicate these patients’ ability to adapt and adhere to the protective measures recommended to prevent infection (such as hand washing, wearing masks, social distancing or mandatory quarantine). Lower awareness to the possible consequences of the COVID-19, paired with the absence of insight to a possible relapse or aggravation of previous symptoms exposes this population to a greater risk for severe clinical outcomes [11, 12].

It is also important to stress that a great amount of people with psychiatric illness rely on regular outpatient visits for evaluation and access to prescription medication, as well as home visits. Once again, nationwide regulations on circulation and mandatory quarantine may have resulted in these visits becoming more difficult to attend to. All evidence cited above could lead us to foresee an increase in psychiatric service hospitalizations associated with the emergence of SARS-CoV-2. However, a study conducted during the first months of lockdown due to the COVID-19 in the Lombardia region found a decrease in the number of admissions in psychiatric wards, with a clear reduction for all diagnostic groups except for anxiety disorders and a longer median length of hospitalization, in comparison to admissions in the same wards during 2019 [14]. Another study which concerned both the Lombardia and Lazio regions in Italy also confirmed a reduction in psychiatric admissions during the COVID-19 lockdown (i.e., March 1-April 30, 2020), with no significant differences between voluntary versus compulsory admissions during the investigated time periods [15]. Similar conclusions were observed in a study performed in Northern India, which showed a reduction in the number of admissions to psychiatric wards during the pandemic period (March 23-September 22, 2020) with a predominance of withdrawals due to psychoactive substance abuse and noncompliance to treatment [16].

The aim of this study was to try to understand if the pandemic context altered the characteristics of the psychiatric hospital admissions of one of the largest psychiatric hospitals in Portugal and to reflect on the clinical care and access to mental health services available at the time.

## Methods

To begin to fully understand how COVID-19 may have affected mental health and help-seeking behaviours of the population in general and psychiatric patients in specific, the authors examined patterns of admissions of patients to acute psychiatric wards in a major psychiatric hospital in Lisbon, Portugal—Centro Hospitalar Psiquiátrico de Lisboa (CHPL). Two time periods were analyzed: the COVID-19 period (from March 11^th^, 2020 to March 10^th^, 2021) and the same time period 1 year previously (pre-COVID-19 period: March 11^th^, 2019 to March 10^th^, 2020). It is important to state that March 11, 2020 was the first day of lockdown measures in Portugal, after confirmation of the first 2 cases of infection with SARS-CoV-2 in March 2. These measures were sustained at least until the end of April, with progressive deconfinement measures from that period on.

During both time periods, variables like age, gender, type of admissions—voluntary versus compulsory ones, the latter being applied, according to the Portuguese Mental Health Law [17], to patients with severe decompensated mental illness who are at risk for harmful behaviour and refusal of treatment (and therefore may be assumed as an indirect measure for severity of the disorder)—time period between previous psychiatric hospitalization and current one, length of admission, readmissions (counted as new psychiatric admissions at the hospital on the 120 days upon discharge) and primary diagnosis at discharge were analyzed retrospectively from hospital records. Diagnoses were clustered and analyzed based on broader diagnostic categories available in the International Classification of Diseases 10th Revision (ICD-10) [18].

CHPL is constituted by at least 6 acute psychiatric wards, admitting patients of all ages and also including a unit solely focused on the treatment of patients in transition from Child Psychiatry to Adult Psychiatry. Patients from 15–25 years old are committed and observed, from 15–17 years, by Child Psychiatrists, and from 18 years old upwards, by Adult Psychiatrists. The data of patients between 18–25 years old admitted to this ward were separately analyzed. Being a more fragile group of patients, in which interpersonal relationships with friends and close relatives assume a bigger role in their stability, they may also be considered as more prone to mental health issues deriving from the general lockdown and school closures [19]. For that reason, these patients were additionally studied in separate, for the same variables.

### Data collection

Upon approval from the hospital’s ethical and scientific committees, the authors were given the anonymized dataset from the hospital, concerning the requested variables. Since identification of single patients was not possible, no consent form was needed or demanded.

### Statistical analysis

The statistical analysis was performed using SPSS 26.0 (IBM, USA). Quantitative variables were tested for normality using Shapiro Wilk test. Comparisons between groups were performed using Mann–Whitney U. The Chi-Square Test of Independence was used to determine association between categorical variables. When more than 20% of cells have expected frequencies lower than 5, Fisher's exact test was used. Significance level was established at 0.05.

Two different populations in the statistical analysis were considered: the total number of patients (N = 3123, 1845 from the pre-COVID-19 period and 1278 on the COVID-19 one) and those aged between 15 to 25 years (N = 388 during control period and N = 268 during the COVID-19 period), which were committed to a transition clinic at work within our hospital,

The following variables were calculated: median age, prevalence of each gender, compulsory treatment, median admission period, number of patients with previous admissions and median of days since those admissions, number of patients who had readmissions (measured as another psychiatric admission in the 120 days after discharge), median days between discharge and new hospitalizations, and different clusters of diagnosis in each sample, according to the sub-chapters of the ICD-10:

- ● Mental disorders due to known physiological conditions (F00-09)
- ● Mental and behavioural disorders due to use of alcohol (F10)
- ● Mental and behavioural disorders due to use of other psychoactive substances (F11-19)
- ● Schizophrenia, schizotypal and delusional disorders (F20-29)
- ● Bipolar disorder (F30-31)
- ● Depressive disorder (F32-34)
- ● Neurotic, stress-related and somatoform disorders (F40-48)
- ● Disorders of adult personality and behaviour (F60-69)
- ● Intellectual disabilities (F70-79)
- ● Others (since the number of other diagnoses were very small overall)

The main purpose of this analysis was to observe the differences between the pre- and the COVID-19 period group.

Additionally, the authors calculated the percentage reduction of admissions between the two periods, regarding the overall number and the diagnostic clusters, in order to understand if all types of diagnoses presented the same variation in admissions.

Finally, regarding compulsory admissions, the frequency of each cluster of diagnosis was compared between pre- and COVID-19 periods.

## Results

### Characteristics of all admitted patients

The final analytic cohort for the sample of hospitalized patients included 1845 patients in the pre-COVID-19 period, and 1278 on the COVID period. Table 1 reports descriptive characteristics of the overall sample, such as median age, gender, prevalence of compulsory treatment, duration of admission, frequency of patients with previous admissions (and median of days since then) and frequency of readmissions in the following 120 days (and median days to these readmissions).

**Table 1:** Characteristics of the sample of 25 + year-old patients admitted to psychiatric wards during the pre- and the COVID-19 period. Y, year; IQR, interquartile range; d, days

	Pre-COVID-19 period (n = 1845)	COVID-19 period (n = 1278)	**p value**
Age, y, median (IQR)	43 (28–56.5)	45 (28–59)	**0.026**^**a**^
Sex, female, n (%)	862 (46.7)	630 (49.3)	0.157^b^
Compulsory admissions, n (%)	494 (26.8)	425 (33.3)	** < 0.001**^**b**^
Duration of admission, d, median	15	16	** < 0.001**^**a**^
Previous admissions, n (%)	905 (49,1)	674 (52.7)	**0.043**^**b**^
Time period from last admission, d, median	261	315	0.181^a^
Psychiatric admissions in the following 120 days, n (%)	299 (16.2)	218 (17.1)	0.529^b^
Psychiatric admissions in the following 120 days, d, median	29	25	0.056^a^

^**a**^Mann-Whitney U test

^b^Chi-Square Test of Independence

Total number of admissions was lower during the COVID-19 period, with less 567 hospitalizations during the COVID-19 period (which corresponds to a reduction of 30.7% of psychiatric admissions during this period of time). The median age of the patients was 43 years old in the control period and 45 years old during the COVID-19 year.

Between the two groups, statistical but small differences were found regarding the age of patients (with older patients being admitted during the COVID-19 period), duration of admission (slightly higher in the COVID-19 period, with a median hospitalization stay of 16 days) and the number of previous admissions (with a small increase of the percentage of re-admitted patients during the COVID-19 period, indicating the hospitalization of patients with pre-existing psychiatric illness). Regarding the frequency of compulsory admissions, there was a slight increase in the percentage of patients being involuntarily admitted during the COVID-19 period (33.3% versus 26.8% pre-COVID-19). No significant differences were found regarding gender, number of days since the last admission, frequency of readmissions or the number of days until these readmissions. Multiple comparisons only found relevance for the duration of admissions and number of compulsory admissions.

### Diagnostic clusters of hospitalized patients

As Table 2 shows, the majority of hospitalizations was due to Schizophrenia, schizotypal and delusional disorders (F20-29) in both groups, corresponding to 28.6% of all admissions during the control period, with an increase to 35.5% of admissions during the COVID-19 period. Depressive disorder (F32-34) diagnoses showed a slight relative increase during the COVID-19 period (from 16.6% of psychiatric admissions pre- to 20.3% during the COVID-19 period), while the relative frequency of Bipolar disorder (F30-31) was similar in both pre- and during COVID-19 periods. It is also important to notice that during the control period there were 258 admissions due to Mental and behavioural disorders due to the use of alcohol (F10), while during the COVID-19 period, these disorders amounted only to 76 hospitalizations. As for Neurotic, stress-related and somatoform disorders (F40-48), Disorders of adult personality and behaviour (F60-69) and Intellectual disabilities (F70-79), there were no significant changes regarding the proportion of hospitalizations during the COVID-19 period. Statistical difference was found regarding the distribution of diagnoses between the two groups.

**Table 2:** Frequency of different diagnoses clusters of 25 + year-old patients admitted to psychiatric wards during the pre- and the COVID-19 period

**Diagnosis**, n (column %)	Pre-COVID-19 period (*n* = 1845)	COVID-19 period (*n* = 1278)	*P* value*	***p* value**
Mental disorders due to known physiological conditions (F00-F09)	76 (4.1)	68 (5.3)	1.0	** < 0.001**^**b**^
Mental and behavioural disorders due to use of alcohol (F10)	258 (14)	76 (5.9)	< 0.001	
Mental and behavioural disorders due to use of other psychoactive substances (F11-19)	101 (5.5)	49 (3.8)	1.0	
Schizophrenia, schizotypal and delusional disorders (F20-29)	528 (28.6)	454 (35.5)	0.075	
Bipolar disorder (F30-31)	313 (17)	228 (17.8)	1.0	
Depressive disorder (F32-34)	307 (16.6)	260 (20.3)	0.016	
Neurotic, stress-related and somatoform disorders (F40-48)	65 (3.5)	35 (2.7)	1.0	
Disorders of adult personality and behaviour (F60-69)	105 (5.7)	53 (4.1)	0.379	
Intellectual disabilities (F70-79)	72 (3.9)	22 (1.7)	0.274	
Others	20 (1.1)	33 (2.6)	0.032	

^b^Chi-Square Test of Independence

^*^Chi-Square Test of Independence. P values were adjusted for multiple comparisons with Bonferroni correction

**Fig. 1:**
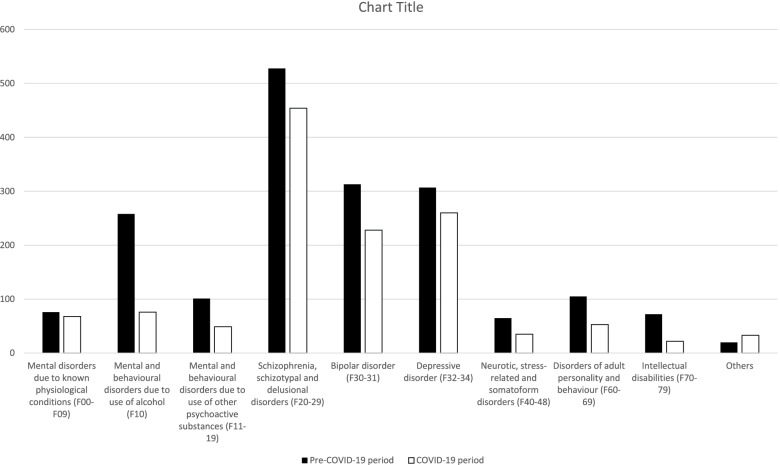
Variance of frequency of diagnosis regarding admission in psychiatric wards during the pre- and the COVID-19 period

Figure 1 shows the discrepancy of admissions between the two groups, regarding their diagnosis.

**Fig. 2:**
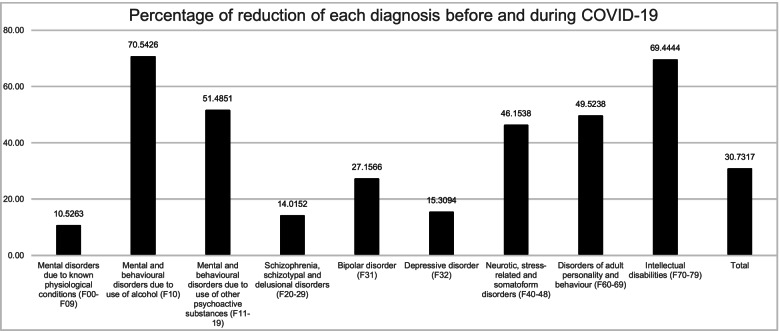
Percentage of reduction of each admission diagnosis between the pre-COVID-19 and COVID-19 groups. The straight line represents the value of the reduction of the overall number of admissions

According to the gathered data, there was a reduction of 30.73% in total admissions between the two groups. However, the proportion of reduction of each diagnosis is very different. As it is clear to understand from Fig. 1 and 2, this reduction was much higher in the patients with Mental and behavioural disorders due to the use of alcohol and other psychoactive substances (F10-19), Neurotic, stress-related and somatoform disorders (F40-48), Disorders of adult personality and behaviour (F60-69) and Intellectual disabilities (F70-79), whereas Mental disorders due to known physiological conditions (F00-09), where Dementias are included, Schizophrenia, schizotypal and delusional disorders (F20-29), Bipolar disorder (F30-31) and Depressive disorder (F32-24) presented a lower reduction of admissions between the two periods.

### Association between diagnostic cluster and compulsory admissions

**Fig. 3:**
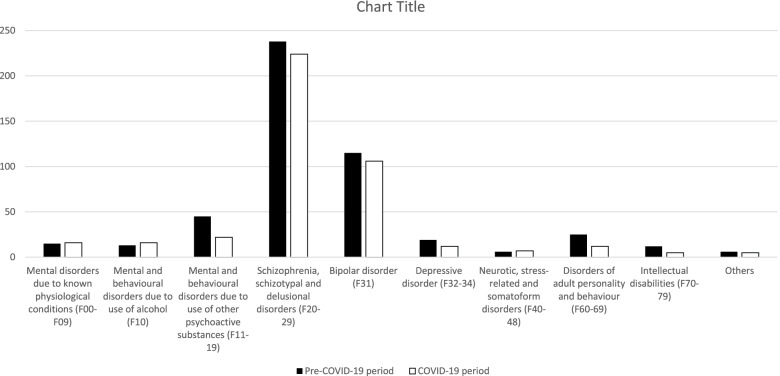
Frequency of compulsory admissions during the pre-COVID-19 and COVID-19 period, according to their diagnosis

Regarding compulsory admissions, even though their relative frequency increased in the COVID-19 group, the distributions of diagnoses were not different between the two groups (p-value = 0.157), with Schizophrenia, schizotypal and delusional disorders (F20-29) and Bipolar disorder (F30-31) corresponding to approximately three-quarters of the total of compulsory admissions. Table 3 and Fig. 3 present these comparisons.

**Table 3:** Frequency of diagnosis of involuntarily admitted patients to psychiatric wards during the pre- and the COVID-19 period

**Diagnosis**, n (column %)	Pre-COVID-19 period (*n* = 1845)	COVID-19 period (*n* = 1278)	***p* value**
Mental disorders due to known physiological conditions (F00-09)	15 (3)	16 (3.8)	0.157^b^
Mental and behavioural disorders due to use of alcohol (F10)	13 (2.6)	16 (3.8)	
Mental and behavioural disorders due to use of other psychoactive substances (F11-19)	45 (9.1)	22 (5.2)	
Schizophrenia, schizotypal and delusional disorders (F20-29)	238 (48.2)	224 (52.7)	
Bipolar disorder (F31)	115 (23.3)	106 (24.9)	
Depressive disorder (F32-34)	19 (3.8)	12 (2.8)	
Neurotic, stress-related and somatoform disorders (F40-48)	6 (1.2)	7 (1.6)	
Disorders of adult personality and behaviour (F60-69)	25 (5.1)	12 (2.8)	
Intellectual disabilities (F70-79)	12 (2.4)	5 (1.2)	
Others	6 (1.2)	5 (1.2)	

^b^Chi-Square Test of Independence

### Characteristics of hospitalized patients from 15–25 years

As mentioned above, one of the acute psychiatric wards in CHPL is solely focused on the treatment of patients in transition from pediatric to adult care. Regarding this group of patients, as shown in Table 4, the final analytic cohort for the sample was 388 admissions during the pre-COVID-19 period and 268 admissions during the COVID-19 period. The total number of admissions was also significantly lower during the COVID-19 period, with less 120 hospitalizations (a decrease of 30,9%). The median age of the patients was 19 years old in the control period and only 18 years old during the COVID-19 period.

**Table 4:** Characteristics of the sample of 15–25 year-old patients admitted to a psychiatric ward during the pre- and the COVID-19 period.. Y, year; d, days

	Pre-COVID-19 period (*n* = 388)	COVID-19 period (*n* = 268)	***p* value**
Age, y, median (IQR)	19 (17–22)	18 (17–22)	0.099^a^
Sex, female, n (%)	193 (49.7)	142 (53)	0.231^b^
Compulsory, n (%)	94 (24.2)	44 (16.4)	**0.010** ^b^
Duration of admission, d, median	13	13	0.747 ^a^
Previous admissions, n (%)	119	102	0.05 ^b^
Time period from last admission, d, median	100	171	**0.036** ^a^
Psychiatric admissions in the following 120 days, n (%)	64	42	0.778 ^b^
Psychiatric admissions in the following 120 days, d, median	24	17.50	0.452 ^a^

^**a**^Mann-Whitney U test

^b^Chi-Square Test of Independence

Prevalence of compulsory hospitalization in patients from 15–25 years was much lower than in the 25 + years sample, with 94 compulsory admissions during the first period and only 44 during the pandemic year. There were no significant differences between groups regarding duration of these admissions, number of previous admissions or readmission rates. Multiple comparison statistics revealed no difference on any variable between these two groups.

### Diagnostic clusters of hospitalized patients between 15–25 years

Within the sample shown in Table 5, the majority of hospitalizations was due to “Depressive Disorder” (F32-34) in both groups, corresponding to a total of 30.9% of all admissions during the pre-COVID-19 period, with a significant increase to 45.5% of total admissions during the COVID-19 period. Schizophrenia, schizotypal and delusional disorders (F20-29) amounted to 24.5% hospitalizations during the control period and only 17.5% during the COVID-19 period. During the pre-pandemic period there were 8 hospitalizations due to Mental and behavioural disorders due to the use of alcohol (F10), with less than half [3] during the COVID-19 period. Intellectual disabilities (F70-79) diagnosis had the biggest downfall during the COVID-19 period, with a decrease of 73.3% of admissions.

**Table 5:** Frequency of different diagnoses clusters of 15–25 year-old patients admitted to psychiatric wards during the pre- and the COVID-19 period

**Diagnosis**, n (column%)	Pre-COVID-19 period (*n* = 388)	COVID-19 period (*n* = 268)	*P* value*	***p* value**
Mental disorders due to known physiological conditions (F00-F09)	0 (0)	1 (0.4)	0.024	**0.001** ^b^
Mental and behavioural disorders due to use of alcohol (F10)	8 (2.1)	3 (1.1)	1.0	
Mental and behavioural disorders due to use of other psychoactive substances (F11-19)	28 (7.2)	14 (5.2)	1.0	
Schizophrenia, schizotypal and delusional disorders (F20-29)	95 (24.5)	47 (17.5)	0.640	
Bipolar disorder (F31)	44 (11.3)	29 (10.8)	1.0	
Depressive disorder (F32-34)	120 (30.9)	122 (45.5)	1.0	
Neurotic, stress-related and somatoform disorders (F40-48)	21 (5.4)	7 (2.6)	0.887	
Disorders of adult personality and behaviour (F60-69)	33 (8.5)	22 (8.2)	1.0	
Intellectual disabilities (F70-79)	30 (7.7)	8 (3.0)	0.520	
Others	9 (2.3)	15 (5.6)	1.0	

^b^Chi-Square Test of Independence

^*^Chi-Square Test of Independence. P values were adjusted for multiple comparisons with Bonferroni correction

## Discussion

The main focus of this study was to evaluate the differences between the characteristics of both samples during a pre-COVID-19 period – March 11, 2019 to March 10, 2020 – and one year after the implementation of lockdown measures in Portugal, due to the COVID-19 pandemic – March 11, 2020 to March 11, 2021 – using data from all acute psychiatric wards in CHPL. During the latter period of time, Portugal was put under rigorous restrictions which included remote work, the closure of schools and other public spaces and, in regards to the health system, at least until end of April 2020, the cessation of all un-emergent activity, such as medical appointments, therapeutic groups and occupational therapy programs. At the time of writing, the present study represented the first study to assess the characteristics of psychiatric admissions in Portugal, during different periods of time, after the emergence of the COVID-19 pandemic.

In line with previous investigations [14–16, 20–23], we found a decrease in total hospitalizations during the COVID-19 period (30.7% less admissions in patients above 25 years old and 30.9% in patients between 15 and 25 years of age). A number of explanations might be proposed for this result. First, the possibility of contagion might have impacted patients’ willingness to seek help for mental health problems, both in outpatient clinics and emergency services. Despite the fact that the absolute number of compulsory admissions was lower during the COVID-19 period in both people under and above 25 years of age, this reduction was lower than the general reduction of admissions (14% reduction in involuntary admissions versus 30.7% reduction in general admissions). This is also represented by an increase in the relative proportion of compulsory admissions during the COVID-19 period (26.8% involuntary admissions during the pre-COVID-period versus 33.3% during the year after emergence of the COVID pandemic). Compulsory admissions are most commonly associated with acute psychiatric manifestations, such as the occurrence of psychotic symptomatology alongside with psychomotor agitation and increased aggressiveness. According to the “hospital avoidance” hypothesis, it makes sense that only those with exuberant psychiatric symptoms were ultimately brought to emergency services, where most compulsory admissions are usually managed. Other studies presented similar results [15, 24, 25].

Another plausible explanation for the reduction of admissions during the first year of the COVID-19 pandemic refers to its severe impact on the health system, including psychiatric wards, which were likely undersupplied during the lockdown. Staff shortages and the inevitable reduction of available beds due patients infected with COVID-19 might have ultimately influenced the hospitalization rates.

There may have also been a tightening of the criteria for psychiatric admission, which could have been equally influenced by the shortage of resources, and also for the unique challenges regarding the hospitalization of individuals infected with COVID-19 in psychiatric units.

It may also be that the reduction of psychiatric hospitalizations is explained by an overall reduction in symptom severity. Actually, evidence has been mixed, with some studies showing that people previously diagnosed with depressive, anxiety or obsessive–compulsive disorders, while having experienced a detrimental impact on their mental health from the COVID-19 pandemic, did not actually show an increase in symptom severity due to the emergence of SARS-CoV-2 infection [26]. On the other hand, studies have found an increase in symptom severity, especially for affective and anxiety disorders during the outbreak of COVID-19, but, probably surprisingly, no changes in the level of OCD or eating disorders [27]. Individuals with affective disorders may also report greater COVID-19-related stress than individuals with schizophrenia spectrum disorders [28].

In both periods of time there seems to be no difference between number of previous admissions or rates of readmissions (after multiple comparisons), but the overall number of patients were hospitalized for only slightly longer periods of time (with a median stay of 15 days before the emergence of the COVID-19 pandemic and 16 days during the COVID-19 period). It is not to exclude, however, the fact that there might be a bias in these conclusions, since the biggest reduction of admissions were observed in patients with usually milder symptoms. If the tightening of criteria for the hospitalization of psychiatric patients was, indeed, a reality, this might explain greater severity of symptomatology in those who are actually admitted, requiring longer hospitalizations during that period. This was also in line with other studies [11, 14, 20, 29]. Medical staff may have also opted for longer admission stays to avoid the risk of re-admission or even for lack of availability of post-acute psychiatric care pathways, due to COVID-19-related restrictions.

It is important to stress the massive decrease in hospitalizations due to “Mental and behavioural disorders due to use of alcohol” (F10) during the COVID-19 period. This could be explained by the fact that admissions for alcohol dependence are mainly programmed and therefore reduced during the pandemic. This poses as potentially problematic, since some evidence shows dramatic increases in harmful alcohol consumption during the first months of the COVID-19 pandemic [30], accompanied by an increase in alcohol-related emergencies, including alcohol withdrawal, withdrawal-related suicides, methanol toxicity and alcohol-related motor vehicle accidents [31]. If this trend also translates into the Portuguese reality, it means that these patients were not adequately attended to during the first year of pandemic.

The most prevalent diagnostic cluster in the sample of 15–25 year old patients was “Depressive disorders” (F32-34), with an actual increase in the frequency of this diagnosis, both in absolute number but also in relative frequency (from 30.9% to 45.5% of the admissions during the pandemic). This may hint at this population’s higher vulnerability to stress and anxiety, paired with less coping skills to handle measures which were implemented during the pandemic (such as school closures or not being able to spend time with their friends) [31]. However, because individual patient files’ weren’t accessed, the authors cannot understand if these admissions due to depressive symptoms were related to any kind of COVID-related stressors, like other studies suggest [14, 31].

An important limitation to our study is the unavailability of studies of secular trends, accompanied by the fact that COVID infections could actually already be present in the Portuguese population before March 2020. The study only pertains to one location, which prevents us to draw conclusions related to nationwide variations in mental health-related hospitalizations. Although our sample represented a total of 1845 and 1278 hospitalizations during the control period and the COVID-19 period, respectively, the wide variety of diagnosis forced us to cluster most of them in certain diagnostic groups. This may also have worked as a limitation to our study as, for example, within the “Mental disorder due to use of other psychoactive substances”, a lot of the patients were diagnosed with polysubstance use, which prevents us from studying which psychoactive substance had the most increase in usage. It is fundamental to stress that correlation does not equal causation, which means further research and evidence is needed to draw conclusions.

This study allowed an evaluation of the hospital’s response during the pandemic. The optimization of our approach to SMI patients and the prediction of future challenges are ahead. It is possible that an increase in future diagnosis of affective and anxiety disorders, suicidality and substance use disorders is due. Only further research would be able to help in the understanding of the long-term effects of this pandemic on severe mental health patients, as well at the efficacy of the institution’s response, in line with the work of Hawryluck L et al. in 2003 [2]. However, since there’s no direct access to patients’ files, the authors will never be able to understand if this pattern of admissions is actually COVID-related.

## Conclusions

The COVID-19 pandemic has undoubtedly imposed a challenging crisis in the previous prevention-and-intervention health care model. It becomes clear there is a need for rapid identification of at-risk groups for psychological and psychiatric complications, which requires proper screening methods, in-time referral and the promotion of early and targeted intervention.

Further research is needed to recognize in which way has the COVID-19 pandemic affected patterns of psychiatric hospital admissions. The authors believe that some consequences might only be acknowledged through the study of this population of patients in the next few years.

## Data Availability

The data supporting the findings of this study are available from Centro Hospitalar Psiquiátrico de Lisboa, but restrictions apply to the availability of these data, which were used under license for the purpose of the study, and so are not publicly available. However, these data are available to the authors and should be consulted under reasonable request and permission of Centro Hospitalar Psiquiátrico de Lisboa. This access should be previously requested via e-mail to the corresponding author.

## Abbreviations

CHPL: Centro Hospitalar Psiquiátrico de Lisboa
COVID-19: 2019 Coronavirus Disease
ICD-10: International Classification of Diseases, 10^th^ Revision (World Health Organization)
OCD: Obsessive–compulsive disorder
SARS-CoV-2: Severe acute respiratory syndrome coronavirus 2
